# The Success of Fabrication of Pure SmFe_2_ Phase Film with Outstanding Perpendicular Magnetic Anisotropy

**DOI:** 10.3390/ma17092027

**Published:** 2024-04-26

**Authors:** Shijie Liao, Fang Wang, Hui Shen, Jian Zhang

**Affiliations:** 1 School of Materials Science and Engineering, Shanghai Institute of Technology, Shanghai 201418, China; liaoshijie970907@163.com; 2 CAS Key Laboratory of Magnetic Materials and Devices, Ningbo Institute of Materials Technology and Engineering, Chinese Academy of Sciences, Ningbo 315201, China; 3 Zhejiang Province Key Laboratory of Magnetic Materials and Application Technology, Ningbo Institute of Materials Technology and Engineering, Chinese Academy of Sciences, Ningbo 315201, China; 4 School of Materials and Chemical Engineering, Ningbo University of Technology, Ningbo 315211, China; wangfangch@nbut.edu.cn

**Keywords:** magnetic films, perpendicular magnetic anisotropy, SmFe_2_

## Abstract

This study used DC magnetron sputtering technology to fabricate Sm-Fe films and systematically investigated the phase transition behavior of Sm-Fe films with different Fe ratios. It was found that at higher Fe content, the films consisted of Sm_2_Fe_17_ or SmFe_7_ phases; as Fe content decreased, the films were mainly composed of SmFe_3_ or SmFe_2_ phases; at higher Sm content, the films primarily consisted of Sm phase. Sm is prone to volatilization at high temperatures, so Ta was used as a capping layer to effectively suppress Sm volatilization, successfully synthesizing pure SmFe_2_ phase films at a nearly 1:2 ratio. The magnetic properties and magnetostrictive behavior of the SmFe_2_ films were investigated, revealing that pure-phase SmFe_2_ films exhibit good perpendicular magnetic anisotropy and magnetostriction properties. The larger stress along the perpendicular-to-film direction, resulting from the absence of substrate-induced constraints, contributes to the excellent perpendicular magnetic anisotropy of the films. This study successfully synthesized pure-phase SmFe_2_ films and discovered a new method for fabricating perpendicularly anisotropic films. The research findings are of great significance for the efficient synthesis of desired films with high phase formation temperatures containing volatile elements.

## 1. Introduction

Magnetic anisotropy is a key characteristic of magnetic materials and plays an important role in various applications, such as spin electronic devices [1,2,3,4], microelectromechanical systems [5], magnetic nanosensors [6], magnetic storage [7], and magnetic recording [8,9]. In magnetic films, the easy magnetization direction is often preferentially oriented within the plane, making it difficult to obtain films with perpendicular magnetic anisotropy, which severely limits the development of high-performance functional devices [10,11]. Epitaxial growth is one effective method for fabricating films with perpendicular anisotropy [12]. However, the perpendicular anisotropy of epitaxial-grown films is influenced by various factors, such as growth conditions, crystal structure, lattice matching, and film thickness, among others [13,14].

Rare-earth transition metal intermetallic compound films exhibit excellent magnetic properties and have been widely used in magnetic microelectromechanical systems, among other fields [15,16]. Among the various rare-earth transition metal intermetallic compound films, Sm-based films such as Sm-Co and Sm-Fe have attracted considerable attention due to their excellent intrinsic magnetic properties. SmCo_5_ and Sm_2_Co_17_ in the Sm-Co system are important magnetic phases. SmCo_5_ exhibits high coercivity and saturation magnetization, with coercivity reaching 15–35 kOe and a maximum energy product of 18–20 MGOe [17]. It also possesses a certain degree of temperature resistance [18]. However, its cost is higher due to the use of the rare element cobalt. On the other hand, Sm_2_Co_17_ outperforms SmCo_5_ in terms of saturation magnetization, with a higher maximum energy product of 27 MGOe, but it is more expensive and temperature-sensitive [17]. Sm_2_Co_17_ and SmCo_5_ phases can be synthesized using physical or chemical methods to produce magnetically anisotropic permanent magnetic materials. Yue et al. successfully synthesized anisotropic SmCo_5_ nanochips with tailored morphologies by designing special-shaped hydroxide precursors. These nanochips exhibited a coercivity of 19.3 kOe and a maximum energy product of 14.4 MGOe [19]. Kumari et al. achieved high magnetic anisotropy in composite materials by chemically synthesizing a combination of soft magnetic Co phase and hard magnetic Sm-Co phase [20]. Sm-Fe films exist in multiple phases, including the amorphous phase, SmFe_2_, SmFe_3_, and Sm_2_Fe_17_, etc. Compared to the SmFe_2_ phase, both the SmFe_3_ and Sm_2_Fe_17_ phases demonstrate higher saturation magnetization and coercivity (SmFe_3_: Ms = 80 emu/g, Hc = 3100 Oe; Sm_2_Fe_17_: Ms = 100 emu/g, Hc = 680 Oe) [21,22,23,24]. In contrast, the reported saturation magnetization and coercivity of the SmFe_2_ phase are 59.8 emu/g and 300 Oe, respectively [25,26]. Notably, among these phases, SmFe_2_ demonstrates exceptional magnetostrictive properties. Due to the presence of multiple phases in Sm-Fe films, it is challenging to obtain pure phase SmFe_2_. Wang et al. discovered that thermal sputtering and heat treatment processes can lead to the formation of SmFe_2_ phase, but they also observed the presence of α-Fe, Sm, oxides, and other impurity phases [27]. Yoon et al. found that Sm-Fe films can be transformed into SmFe_2_ through high-temperature annealing, but Fe precipitation occurs as well [28]. However, current research on Sm-Fe films mostly focuses on the synthesis of simple amorphous films. For instance, Honda et al. fabricated cantilever actuators and movable devices composed of amorphous Tb-Fe and Sm-Fe films, demonstrating characteristics such as large deflection and wireless driving. The magnetostriction coefficient of SmFe films under a 1000 Oe magnetic field ranges from −250 ppm to −300 ppm, and they exhibit in-plane magnetic anisotropy [29,30]. Quandt and Seemann prepared amorphous Tb-Dy-Fe and Sm-Fe films and designed and manufactured actuators for finite element analysis, demonstrating great potential for application in microsystems. The SmFe films exhibit an in-plane magnetic easy axis, and the magnetostriction coefficient reaches a maximum of around −350 ppm under a bias voltage of 120 V and a 6000 Oe magnetic field [31]. Currently, the coercivity of the SmFe amorphous phase can reach 265 Oe [32]. Therefore, the challenge lies in preparing SmFe_2_ films with high magnetic performance, and successfully synthesizing SmFe_2_ films with perpendicular magnetic anisotropy is also an intriguing research topic.

In this work, we successfully prepared SmFe_2_ films with perpendicular magnetic anisotropy and high magnetic performance using DC magnetron sputtering. We systematically investigated the phase formation behavior of SmFe films with different Fe composition ratios. We found that at higher Fe content, the films consist of Sm_2_Fe_17_ or SmFe_7_ phases, while a decrease in Fe content enables the formation of the SmFe_2_ phase. In the absence of a capping layer, Sm has high volatility at elevated temperatures, resulting in the formation of SmFe_7_ or SmFe_3_ phases under compositions close to those of SmFe_2_. To ensure the formation of the SmFe_2_ phase, the high-temperature evaporation of Sm can be compensated by appropriately increasing the Sm content in the stoichiometric ratio. Moreover, since Ta exhibits good ductility and resistance to oxidation, it is chosen to be deposited as a covering layer on SmFe films. By depositing a Ta capping layer, the volatilization of Sm is effectively suppressed, ensuring the high-temperature phase formation of near-stoichiometric SmFe_2_ polycrystalline films. Finally, we investigate the magnetic and magnetostrictive properties of the SmFe_2_ polycrystalline films. It is found that the films exhibit small stress in the parallel film plane direction due to substrate constraint but large stress in the perpendicular film plane direction, resulting in good perpendicular magnetic anisotropy.

## 2. Materials and Methods

The Sm-Fe film is prepared using the DC magnetron sputtering technique, with a sputtering chamber vacuum of 2–3 × 10^−6^ Pa. Both Fe (99.99%) and Sm (99.99%) targets are used for sputtering; the Sm-Fe film is deposited using the co-sputtering method. The film thickness is measured using a scanning probe microscope (SPM, Dimension-ICON, Bruker, Marlborough City, MA, USA) according to the thickness of the deposited film after a certain period of time, and the deposition rate of the film is calculated. The elemental composition of the Sm-Fe film is adjusted based on the sputtering power of the Sm and Fe targets. The Sm-Fe film, with a thickness of approximately 1 μm, is deposited on a 500 μm thick (100) Si substrate, with a tantalum (Ta) layer serving as both a buffer and a cover layer. Two types of films are prepared: SmFe_x_(T)/Ta(AA) (x = 0.25–1.6/1.9, T = 500 °C/700 °C) and SmFe_x_(T)/Ta(BA) (x = 1.5–9, T = 250 °C–700 °C), where x represents the Fe composition, T indicates the deposition temperature, AA indicates the deposition of a Ta cover layer after annealing, and BA indicates the deposition of a Ta cover layer before annealing. The annealing time is 15 min to 1 h. Argon gas (5N) is used as the sputtering gas at a pressure of 0.8 Pa.

The phases of the Sm-Fe film are analyzed using an X-ray diffraction analyzer (XRD, D8 ADVANCE, Bruker, Bremen, Germany). The magnetic properties of the Sm-Fe film are measured using a vibrating sample magnetometer (VSM, Lakeshore 7410, Lakeshore, Carson, CA, USA). The surface and cross-sectional morphology of the Sm-Fe film are characterized using scanning electron microscopy (SEM, G300, ZEISS, Jena, Germany) with energy-dispersive X-ray spectroscopy (EDS), and the cross-sectional morphology is observed using hand-broken samples. The magnetostrictive coefficient of the Sm-Fe film is measured by a material magnetostrictive characteristic measuring instrument (BKT-2600, BEIJING SHINCO TEST SCIENCE AND TECHNOLOGY CO. LTD, Beijing, China), with a maximum parallel magnetic field of ΔH_∥_ = 1 T.

## 3. Results and Discussion

### 3.1. XRD Analysis

**Figure 1:**
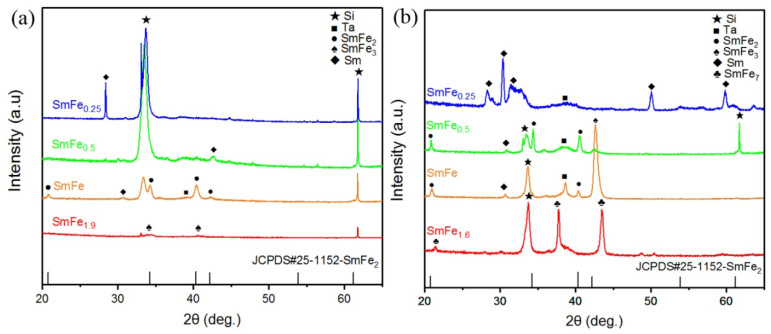
(**a**) XRD patterns of SmFe_x_ (x = 0.25–1.9) films deposited at 500 °C and annealed at 700 °C for 1 h (with Ta capping layer after annealing); (**b**) XRD patterns of SmFe_x_ (x = 0.25–1.6) films deposited at 700 °C (with Ta capping layer after high-temperature deposition).

The investigated study focuses on high-temperature deposition, followed by subsequent annealing, and the phase formation behavior of Sm-Fe films with varying Fe composition ratios x by depositing a Ta protective layer on top after high-temperature annealing. Table 1 presents the crystallographic basic information of potential phases existing in Sm-Fe films. The peaks at 2θ = 33°, 61° and 70° are from the Si substrate [33]. Figure 1a shows the XRD patterns of SmFe_x_(500 °C)/Ta(AA) films annealed at 700 °C for composition ratios x = 0.25–1.9. It can be observed that the SmFe_3_ phase forms at composition x = 1.9, while the SmFe_2_ phase, along with a small amount of Sm phase, can be formed for the film of x = 1, indicating the effectiveness of the Ta coating layer due to the absence of Sm oxide phase. Moreover, decreasing the Fe composition ratio to x = 0.5 or 0.25 results in the disappearance of the SmFe_2_ phase, with the Sm-Fe film primarily composed of the Sm phase. Figure 1b presents the XRD patterns of SmFe_x_(700 °C)/Ta(AA) films deposited at high temperatures without subsequent annealing treatments for x = 0.25–1.6. For the composition x = 1.6, the film is mainly composed of the low-Sm-content SmFe_7_ phase. Further reducing the Fe content to a composition ratio of 1:1 in Sm-Fe films leads to the appearance of the SmFe_2_ phase, along with the presence of SmFe_3_ and Sm phases. When the Fe content decreases to a composition ratio of x = 0.5, the dominant phase is SmFe_2_, with a small amount of Sm phase also present. Finally, at the lowest Fe composition ratio of x = 0.25, the SmFe_2_ phase disappears, and the film is mainly composed of the Sm phase. From the above experiments, it can be found that the composition ratio required for the formation of the SmFe_2_ main phase is significantly higher than the stoichiometric ratio of SmFe_2_, indicating the high volatility of Sm during the thermal deposition and annealing processes. Additionally, 700 °C deposition of the film samples with a composition ratio of SmFe_0.5_ leads to the formation of the SmFe_2_ main phase, while films with the SmFe composition ratio deposited at 500 °C and subsequently annealed at 700 °C exhibit the formation of the SmFe_2_ main phase. This suggests that Sm has a higher volatility during high-temperature deposition, requiring a higher Sm composition ratio for the formation of the SmFe_2_ phase.

**Figure 2:**
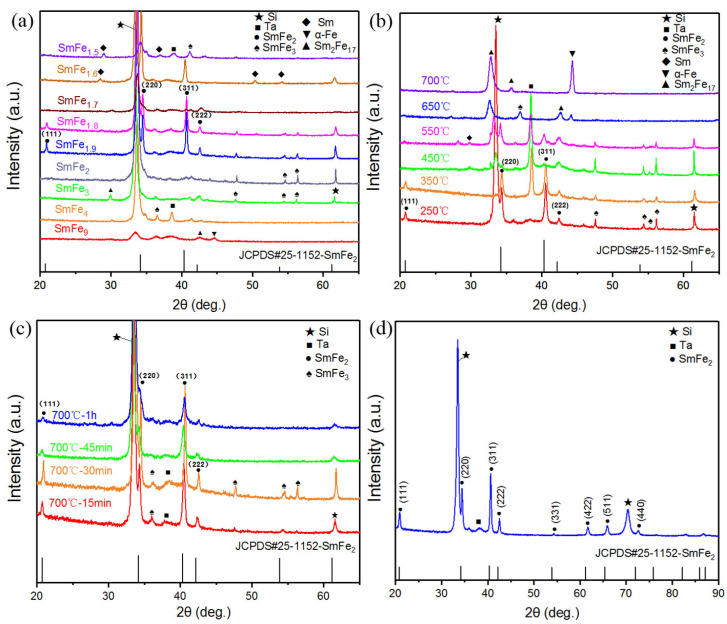
(**a**) XRD patterns of films deposited with SmFe_x_ (x = 1.5–9) layer at 300 °C, followed by deposition of a 50 nm Ta capping layer and then annealing at 700 °C for 0.5 h. (**b**) XRD patterns of films deposited with SmFe_2_ layer at different temperatures (250 °C to 700 °C), followed by deposition of a 50 nm Ta capping layer and then annealing at 700 °C for 0.5 h. (**c**) XRD patterns of films deposited with SmFe_2_ layer at 300 °C, followed by deposition of a 50 nm Ta capping layer and annealing at different times (15 min to 1 h) at 700 °C. (**d**) XRD patterns of films were deposited with SmFe_1.9_ layer at 500 °C, followed by deposition of a 50 nm Ta capping layer and then annealing at 700 °C for 1 h.

In order to mitigate the negative effects of volatile Sm during high-temperature annealing on the formation of the SmFe_2_ phase, a Ta capping layer was applied to the Sm-Fe film before high-temperature annealing to prevent Sm volatilization. The phase transition behavior of this kind of Sm-Fe films with different composition ratios, deposition temperatures, and annealing times was investigated. Figure 2a presents the XRD diffraction patterns of the SmFe_x_(300 °C)/Ta(BA) film with a composition of x ranging from 1.5 to 9, annealed at 700 °C for 0.5 h. It can be observed that when the composition is x = 9, the film is mainly composed of Sm_2_Fe_17_ with an additional strong α-Fe diffraction peak. Further reducing the Fe composition to x = 4 results in the disappearance of the α-Fe phase [33]. For x = 3 and 4, the film is mainly composed of Sm_2_Fe_17_ and SmFe_3_ phases. At x = 2, the Sm_2_Fe_17_ phase vanishes, and the Sm-Fe film is mainly composed of SmFe_2_ and SmFe_3_ phases. At x = 1.9, the film can form the SmFe_2_ phase, but a small amount of the SmFe_3_ phase is also present. When x is further reduced to 1.5 and 1.6, Sm diffraction peaks appear, indicating an excessive addition of Sm. The experimental results reveal that the composition range for the formation of the main SmFe_2_ phase in the film, with the Ta capping layer applied before annealing, is x = 1.8–2. Within this composition range, the Sm content in the film is much lower compared to the film with the Ta capping layer applied after annealing, affirming the effectiveness of the Ta capping layer in suppressing Sm volatilization.

Based on the determination of the composition range for the formation of the main SmFe_2_ phase from Figure 2a, Sm-Fe films with different deposition temperatures were prepared to find the optimal deposition temperature. These films were then subjected to high-temperature annealing with a Ta capping layer applied before annealing. Figure 2b shows the XRD patterns of SmFe_2_(250 °C–700 °C)/Ta(BA) films deposited at various temperatures. Within the temperature range of 250 °C–550 °C, the films can form the main SmFe_2_ phase with a small amount of SmFe_3_ phase. Notably, the films deposited at 250 °C and 350 °C exhibit a lower proportion of SmFe_3_ phase compared to those deposited at 450 °C and 550 °C. When the deposition temperature is increased to 650 °C, the α-Fe phase, which had disappeared for x = 4, reappears. Moreover, when the deposition temperature is raised to 700 °C, the intensity of the α-Fe diffraction peak increases [27], indicating an elevated Sm volatilization rate. Therefore, only at lower deposition temperatures can the high-Sm-containing SmFe_2_ and SmFe_3_ phases form.

Figure 2c displays the XRD patterns of the SmFe_2_(300 °C)/Ta(BA) film with a composition of x = 2, annealed at 700 °C for different durations. For annealing times of 15 min–30 min, the film is mainly composed of SmFe_2_ and SmFe_3_ phases. As the annealing time reaches 45 min, a pure phase of polycrystalline SmFe_2_ film is formed. Compared to the 45-minute annealed sample, the film annealed for 1 h should exhibit a larger grain size in the SmFe_2_ phase.

Based on the experimental results shown in Figure 2a–c, the conditions for the formation of the SmFe_2_ phase in the film are determined to be a composition ratio within x = 1.8–2, a deposition temperature within 250 °C–550 °C, and an annealing time within 45 min–1 h. As a further example, a SmFe_1.9_ film with a composition ratio of x = 1.9 was prepared and annealed at 700 °C for 1 h, as shown in Figure 2d. It can be observed that a pure phase of polycrystalline SmFe_2_ film is formed in this sample. Our research results from Figure 1 and Figure 2 demonstrate that the deposition of a Ta capping layer effectively prevents Sm volatilization. By adjusting the deposition temperature and annealing time close to the proper composition ratio for the SmFe_2_ phase, we have successfully fabricated a single-phase polycrystalline SmFe_2_ film.

### 3.2. Magnetic Performance Analysis

**Figure 3:**
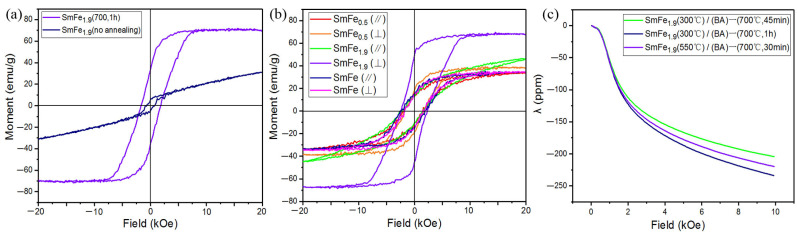
(**a**) Magnetic hysteresis loop of the SmFe_1.9_(500 °C)/Ta(BA) film samples in the perpendicular film plane direction, both as-deposited and annealed at 700 °C for 1 h (SmFe_1.9_(500 °C)/Ta(BA)–(700 °C, 1 h)); (**b**) Magnetic hysteresis loops of the SmFe_1.9_(500 °C)/Ta(BA) film annealed at 700 °C for 1 h, as well as the SmFe(500 °C)/Ta(AA) film annealed at 700 °C for 1 h and the as-deposited SmFe_0.5_(500 °C)/Ta(AA) film sample in parallel (//) and perpendicular (⊥) directions; (**c**) Magnetostrictive coefficient chart of the SmFe_1.9_(500 °C)/Ta(BA) film annealed at different times at 700 °C.

We have investigated the magnetic properties of the prepared Sm-Fe film samples. Figure 3a shows the magnetic hysteresis loop of the SmFe_1.9_(500 °C)/Ta(BA) film samples of as-deposited and annealed at 700 °C for 1 h, respectively, along the direction perpendicular to the film surface. Compared with annealed samples, the as-deposited sample exhibits a low coercivity of 641 Oe and a saturation magnetization of 30.9 emu/g, possibly due to the poor crystallinity of the as-deposited film. After annealing at 700 °C, the SmFe_1.9_(500 °C)/Ta(BA) film transforms into a polycrystalline SmFe_2_ film, resulting in higher coercivity and saturation magnetization. The coercivity reaches 2427 Oe, while the saturation magnetization and residual magnetization are 68.6 emu/g and 48.4 emu/g, respectively. These values are significantly higher than those previously reported, and compositions with ratios close to SmFe_2_ films can achieve a maximum coercivity of 650 Oe [32,33,34,35,36]. Samata et al. reported a saturated magnetization of 59.8 emu/g for polycrystalline grown using the self-flux method, while the saturated magnetization of the films we prepared was 68.6 emu/g, slightly higher than their reported value [25]. Figure 3b shows the magnetic hysteresis loops of the SmFe_1.9_(500 °C)/Ta(BA)–(700 °C, 1 h), SmFe(500 °C)/Ta(AA)–(700 °C, 1 h), and SmFe_0.5_(500 °C)/Ta(AA) film samples along the parallel and perpendicular directions to the film surface. The saturation magnetization along the perpendicular direction is higher than that along the parallel direction for all these samples. This is because the different preparation conditions lead to the formation of the SmFe_2_ main phase in all the Sm-Fe films, and the excellent magnetostrictive properties of the SmFe_2_ phase induce stress in the films under high magnetic fields. The presence of the Si substrate restricts the stress magnitude along the parallel direction, resulting in lower coercivity and saturation magnetization. On the other hand, there is no restriction on stress along the perpendicular direction, resulting in higher values for coercivity and saturation magnetization. According to the Scherrer formula: D = kλ/βcos θ, where k is the Scherrer constant (typically 0.89), λ is the X-ray wavelength, θ is the Bragg diffraction angle, and β is the full width at half maximum of the diffraction peak. The crystallite size SmFe_1.9_(500 °C)/Ta(BA)–(700 °C, 1 h), SmFe(500 °C)/Ta(AA)–(700 °C, 1 h), and SmFe_0.5_(500 °C)/Ta(AA) samples were calculated to be 31.1 nm, 21 nm, and 30 nm, respectively. The significant difference in magnetic properties may be due to the formation of pure SmFe_2_ phase in SmFe_1.9_(500 °C)/Ta(BA)–(700 °C, 1 h) sample, without the non-magnetic Sm phase. In addition, applying a magnetic field along the perpendicular direction generates higher stress, leading to superior magnetic properties compared to other Sm-Fe films. Our results indicate a novel strategy to develop perpendicular anisotropy in films. Figure 3c shows the magnetostrictive coefficient (λ) of the 1:2 phase films prepared at different deposition temperatures and annealing times for SmFe_1.9_(T)/Ta(BA) films. At the same deposition temperature, a longer annealing time results in a larger magnetostrictive coefficient (λ) for the Sm-Fe films. This is possibly due to the larger grain size obtained with longer annealing times, indicating that prolonged annealing enhances the magnetostrictive coefficient of the films. Additionally, the magnetostrictive coefficient of the sample deposited at 550 °C and annealed at 700 °C for 30 min is higher than that of the sample deposited at 300 °C and annealed at 700 °C for 45 min, suggesting that higher temperature deposition and annealing lead to higher purity of the SmFe_2_ phase. However, the maximum magnetostrictive coefficient achieved in the Sm-Fe films we prepared is −234 ppm, which is lower than the values reported in the literature [37,38]. This may be attributed to the high thickness of the Si substrate used for depositing the Sm-Fe films, which affects the magnitude of stress [39].

### 3.3. Morphology Analysis

**Figure 4:**
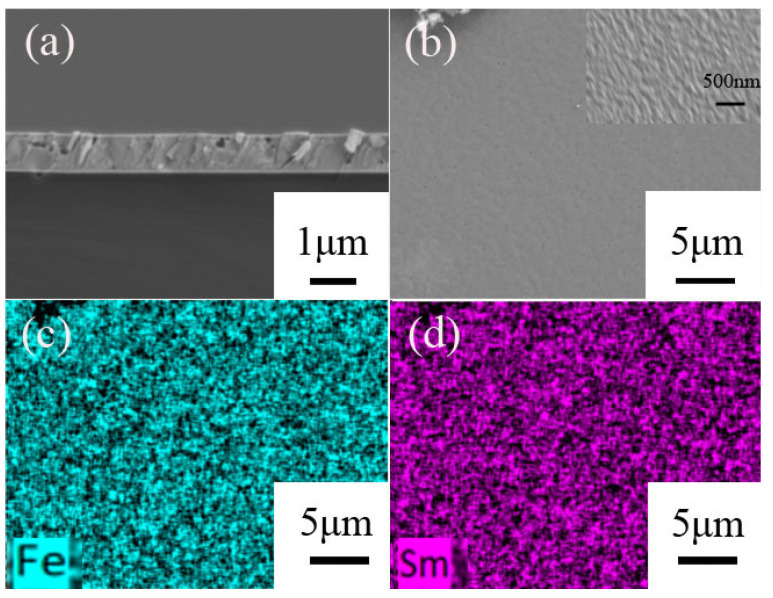
SEM and EDS images of the annealed (700 °C, 1 h) SmFe_1.9_(500 °C)/Ta(BA) film: (**a**) Cross-sectional SEM image of the film; (**b**) Surface SEM image of the film; (**c**) Elemental distribution of Fe in the film surface; (**d**) Elemental distribution of Sm in the film surface.

Figure 4 illustrates the surface and cross-sectional morphology of the film sample SmFe_1.9_(500 °C)/Ta(BA)–(700 °C, 1 h), and the elemental distribution on the surface of the film sample was analyzed using EDS. Examining the cross-section of the film provides information about its density and thickness. Figure 4a presents the SEM image of the cross-section of the film sample, revealing a thickness of approximately 1 μm for the Sm-Fe film, which closely matches the design based on deposition rate. The film contains a small amount of pores of different sizes. Figure 4b shows the SEM image of the film surface, which appears remarkably smooth. The locally enlarged SEM image of the SmFe_2_(550 °C)/Ta(BA) sample annealed at 700 °C for 0.5 h is placed in the upper right corner of Figure 4b. According to the image, the estimated grain size is 135.6 nm, which is larger than the grain size calculated by the Scherrer formula. The possible reason for this is that the deposited Ta coating layer has enlarged the grains. Figure 4c,d display the distribution of Sm and Fe elements on the film surface, demonstrating a relatively uniform distribution throughout the entire surface of the sample. Through quantitative analysis of the Sm and Fe content, it was found that the content of Sm and Fe is 29 at% and 71 at%, respectively, which closely aligns with the compositional ratio of Sm and Fe. This further proves that the deposition of the Ta covering layer effectively prevents the volatilization of Sm during high-temperature heat treatment of the Sm-Fe film.

## 4. Conclusions

This work employs an ultra-high vacuum magnetron sputtering system to fabricate Sm-Fe films and systematically investigate the influence of different Fe content, deposition temperature, and annealing time on the phase formation in Sm-Fe films. Without depositing a Ta layer and performing high-temperature deposition and annealing, the main phase of SmFe_3_ or SmFe_7_ formed at a composition ratio of SmFe_1.9_ or SmFe_1.6_, and the main phase of SmFe_2_ formed only when the Fe content decreased to SmFe or SmFe_0.5_; by depositing a 50 nm Ta layer, the film mainly consisted of Sm_2_Fe_17_ phase and α-Fe at the SmFe_9_ composition ratio, and when the Fe content was lowered to SmFe_3_, it mainly consisted of Sm_2_Fe_17_ and SmFe_3_ phases; further reducing the Fe content to near the stoichiometric ratio of SmFe_2_ (x = 1.9), the main phase of SmFe_2_ formed in the Sm-Fe film. With the deposition of the Ta capping layer before annealing, pure SmFe_2_ phase films (SmFe_1.9_(500 °C)/Ta(BA)–(700 °C, 1 h)) are successfully obtained by depositing at 500 °C and annealing at 700 °C for 1 h. Compared to the main phase SmFe_2_ films prepared without the Ta capping layer, the SmFe_1.9_(500 °C)/Ta(BA)–(700 °C, 1 h) films exhibit a higher magnetostriction coefficient due to the formation of a purer SmFe_2_ phase. The magnetostrictive coefficient is −234 ppm, lower than values reported in some literature, possibly due to the thicker silicon substrate. Consequently, under a magnetic field perpendicular to the film surface, the SmFe_1.9_(500 °C)/Ta(BA)–(700 °C, 1 h) films demonstrate significantly superior magnetic properties in the direction perpendicular to the film surface compared to films with other compositions. This highly perpendicular anisotropic film demonstrates significantly higher coercivity in the perpendicular direction, reaching 2427 Oe, surpassing 650 Oe reported in the literature for films close to the composition of SmFe_2_. The deposition of the Ta capping layer effectively suppresses the volatilization of Sm and facilitates the fabrication of films with desired phases. The success of the fabrication of good perpendicularly anisotropic magnetic films with pure SmFe_2_ phase holds significant research value for the application in functional materials and devices.

## Figures and Tables

**Table 1:** The table shows the space group, crystal structure type, and lattice parameters of different phases according to the JCPDS (Joint Committee on Powder Diffraction Standards) card numbers.

Phase	Space Group	Structure Type	Lattice Constant (Å)
SmFe_7_ (JCPDS#19-0621)	R-3m	TbCu7	a = 8.554 c = 12.441
Sm_2_Fe_17_ (JCPDS#49-1412)	R-3m	Th2Zn17	a = 8.563 c = 12.455
SmFe_3_ (JCPDS#50-1450)	R-3m	PuNi3	a = 5.179 c = 24.795
SmFe_2_ (JCPDS#25-1152)	Fd3m	MgCu2	a = 7.415
Sm (JCPDS#06-0419)	R-3m	一	a = 3.629 c = 26.203
Ta (JCPDS#04-0788)	Im3m	一	a = 3.305

## Data Availability

Data are contained within the article.
